# Oxidative Imbalance in Endometriosis-Related Infertility—The Therapeutic Role of Antioxidants

**DOI:** 10.3390/ijms25126298

**Published:** 2024-06-07

**Authors:** Izabela Dymanowska-Dyjak, Karolina Frankowska, Monika Abramiuk, Grzegorz Polak

**Affiliations:** 1 Independent Laboratory of Minimally Invasive Gynecology and Gynecological Endocrinology, Medical University of Lublin, 20-059 Lublin, Poland; i.dymanowska@gmail.com (I.D.-D.); monika.abramiuk@umlub.pl (M.A.); 2 Student Scientific Association, Independent Laboratory of Minimally Invasive Gynecology and Gynecological Endocrinology, Medical University of Lublin, 20-059 Lublin, Poland

**Keywords:** endometriosis, antioxidants, supplementary treatment

## Abstract

Endometriosis in half of affected women is closely related to problems with fertility. Endometriosis-associated infertility is caused by a wide range of abnormalities affecting the female reproductive tract, from oocyte quality impairment to disturbances in the eutopic endometrium or mechanical abnormalities resulting from disease progression. Since supportive antioxidant therapies, in addition to surgical treatment or assisted reproductive techniques (ARTs), have overall been proven to be effective tools in endometriosis management, the objective of our review was to analyze the role of antioxidant substances, including vitamins, micronutrients, N-acetylcysteine (NAC), curcumin, melatonin, and resveratrol, in endometriosis-related infertility. Most of these substances have been proven to alleviate the systemic oxidant predominance, which has been expressed through decreased oxidative stress (OS) markers and enhanced antioxidative defense. In addition, we demonstrated that the predominant effect of the aforementioned substances is the inhibition of the development of endometriotic lesions as well as the suppression of pro-inflammatory molecules. Although we can undoubtedly conclude that antioxidants are beneficial in fertility support, further studies explaining the detailed pathways of their action are needed.

## 1. Introduction

Multidimensional causes leading to the impairment of a female individual’s ability to conceive render infertility a very complex disorder [1]. Despite years of extending scientific research, the precise determination of the entire range of disturbances underlying this condition often remains challenging [2]. According to the meta-analysis conducted by Nik Hazlina et al., almost half of the general female population may be burdened with various causes of infertility [3]. Researchers show that the diagnosis of endometriosis in infertile women ranges from 30 to 50% of cases. Although these estimates differ, the literature indicates an interplay between these two conditions [4,5].

Endometriosis consists of the abnormal placement of both endometrial glands and stroma, which may appear in the form of ovarian cysts or be implanted on the peritoneal surface, as well as in the organs of the deep pelvis compartment [6,7]. 

Briefly, the mechanisms underlying endometriosis-related infertility can be divided into two main subgroups. On the one hand, endometriosis heightens the occurrence of some anatomical disturbances, including adhesions, tubal patency impairment, or the deterioration of ovarian tissue. On the other hand, this disease affects fertility on the microscopic level due to the destructive character of ectopic tissue [8,9]. Indeed, it fosters a state of a pro-oxidative environment of increased exacerbation to the pelvic cavity, where ectopic lesions most often occur. The main driving force of this highly oxidative state is the growth, accumulation, and following periodical disintegration of pathological endometrial tissue [10]. Such a cascade of processes leads to the release of high amounts of iron and hemoglobin, stimulating reactive oxygen species (ROS) production which, through oxidative imbalance and chronic inflammation, further exerts various detrimental effects on the female reproductive system [10,11,12]. 

To date, there is no single appropriate management method for the treatment of endometriosis-associated infertility, and both surgery and assisted reproductive techniques (ARTs) could be offered [13,14]. Overall, a great amount of scientific evidence suggests the efficacy of some supportive therapies, including a proper diet, supplementation, and physical activity, in endometriosis management [15,16]. Since supplementation particularly concerns products that can modulate the processes underlying the disease, such as inflammation or oxidative stress (OS), it seems crucial to supplement products with antioxidant potential [17]. The multitude of substances exhibiting such properties makes it somewhat difficult to qualify them; however, it can be assumed that several vitamins and microelements, as well as other substances, including melatonin, N-acetylcysteine (NAC), or resveratrol, can act as oxidative balance defenses [18,19,20]. 

So far, the literature has investigated the role of antioxidants in endometriosis; however, they have mainly focused on their role in mitigating the disease-accompanying pain [21,22]. On the other hand, although the role of supplements displaying antioxidant capacity in improving female fertility status has also been discussed, the data referred to the patients with various causes of infertility. The meta-analysis conducted by Showell et al. in 2020 shows that melatonin, coenzyme Q10, NAC, and L-carnitine have promising influences on the clinical pregnancy rate; however, the conclusions are not specific for endometriosis-associated infertility. So far, there is a lack of a qualitative summary of the role of antioxidants in individuals with endometriosis-related infertility [23]. 

Hence, in this review, we summarize the effects of antioxidant supplementation considering such substances as vitamins and micronutrients, melatonin, coenzyme Q10, resveratrol, and NAC on endometriosis-associated infertility.

## 2. Oxidative Stress (OS) as a Cause of Female Fertility Impairment in Endometriosis

There is no doubt that endometriosis is linked not only to the local enhancement but also to the systemic enhancement of the pro-oxidative environment [11]. Years of research on the pathogenesis of endometriosis revealed that affected patients present an altered picture of systemic OS markers [24,25]. Therefore, it can be concluded that the predominance of OS as a cause of endometriosis should form part of the increasingly postulated concept of the systemic character of endometriosis. Thus, endometriosis should be considered a general OS disease [26].

**Figure 1:**
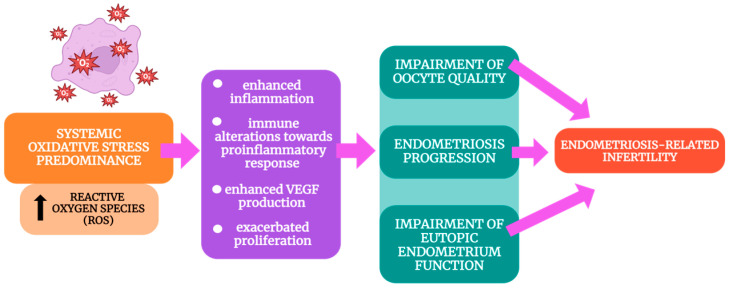
The effects of oxidative stress on fertility in patients with endometriosis based on [27,28].

Although limited studies have been conducted on the detailed mechanisms of OS action in endometriosis-associated infertility, it can be generalized that the excess of ROS results in multi-faceted alterations, including the enhanced production of pro-inflammatory molecules such as interleukine-6 (IL-6), tumor necrosis factor-alpha (TNF-α), or IL-1β, as well as immune shifts toward the predominance of the pro-inflammatory state, alterations in vascular endothelial growth factor (VEGF) production, and increased proliferation through mitogen-activated protein kinase (MAPK) pathway stimulation [27,28]. In turn, these processes contribute to endometriosis progression and the impairment of the quality of oocytes and the eutopic endometrium (Figure 1). As these phenomena can even be observed in patients presenting mild forms of endometriosis, they seem to be responsible for fertility impairment in a significant percentage of affected women [12].

Endometriosis not only deteriorates the eutopic endometrium but also affects the ovaries, which leads to decreased oocyte quality. Overall, the background of alterations in oocyte quality in patients with endometriosis is slightly questionable as researchers have displayed doubtful attitudes about whether the damage caused to the oocytes is primary or occurs as a result of cells’ exposure to the toxic peritoneal fluid environment [29]. Most researchers have indirectly reported OS-related decreased oocyte quality in patients with endometriosis based on associations between increased concentrations of OS markers or reduced values of antioxidant biomarkers in the direct vicinity of oocytes, that is, follicular fluid or granulosa cells, and lower oocyte retrieval, the maturation of oocytes, and in vitro fertilization or pregnancy outcomes [30,31,32]. To date, the potential mechanisms responsible for this state have not been sufficiently investigated. It has been found that an enhanced OS state contributes to the impairment of reproductive functions through the senescence process of granulosa cells [33,34]. The same effects were reported to be the results of granulosa cells’ mitochondrial dysfunction and their potentiated apoptosis [34,35]. Oxidative imbalance was also described as a trigger for some disturbances in the oocytes observed in the endometriosis model, e.g., mitochondrial dysfunction [35].

In addition, endometriosis is also linked to various alterations within the eutopic endometrium, among other hormonal and inflammatory changes, which worsen fertility potential, and OS is perhaps also partially responsible for these changes [29]. Similarly, as in the case of granulosa cells, the exacerbated senescence of the eutopic endometrium in patients with endometriosis was observed [36], and the reports indicate that this process may impair embryo implantation [37]. The state of the enhanced OS has also been mentioned to cause the DNA damage and apoptosis of eutopic endometrial cells, which hypothetically deteriorates the endometrial condition [38,39].

The predominance of an oxidative environment also seems to be responsible for fertility impairment associated with endometriosis progression, such as adhesion formation or poorer parameters of peritoneal fluid [40]. Therefore, these processes impair fertility through the widest range of disturbances. Researchers have revealed that oxidative imbalance has a direct influence on pathways involved in ectopic endometrial cells’ proliferation and invasion [41,42]. In addition, OS has also been described as a factor participating in pathways stimulating angiogenesis, which is critical in the formation of new endometrial lesions [43]. In addition to processes involved in lesion expansion, a direct association between oxidative imbalance and adhesion formation was observed by González-Foruria et al. They found that OS stimulated Notch signaling, which resulted in enhanced fibrosis [44].

## 3. The Impact of Selected Antioxidants on Endometriosis-Related Infertility

### 3.1. Antioxidant Vitamins

Among all vitamins, vitamins C and E are especially vaunted for their antioxidant potential [45]. Indeed, this fact has been reflected in the design of studies on the effects of vitamin supplementation on endometriosis symptoms and development, as a great number of researchers have focused on the combined action of the aforementioned vitamins. So far, several randomized controlled trials (RCTs) including patients with endometriosis, which aimed to evaluate the effects of supplementation with vitamins displaying antioxidant potential on the endometriosis course, have been conducted (Table 1). In general, combined supplementation with vitamin C and vitamin E [46,47,48], as well as a combination of vitamins C, E, and A [49], was capable of affecting the systemic oxidative state of the organism. Such action resulted in decreased serum concentrations of OS markers, such as ROS, malondialdehyde (MDA), or lipid hydroperoxides (LOOHs), and improved values of antioxidant enzymes such as glutathione peroxidase (GPx) or superoxide dismutase (SOD) [46,47,48,49], which proves that these vitamins have protective effects. Only two RTCs, conducted by Mier-Cabrera et al. and Lu et al., assessed the pregnancy outcomes in the conditions of vitamin C or combined vitamin C and vitamin E supplementation, respectively. Although the researchers did not find a direct influence of these compounds on fertility outcomes, there are some noteworthy possible explanations for this situation [48,50]. First, the study by Lu et al. only involved supplementation with vitamin C; therefore, there is a concern about the insufficient isolated action of this vitamin [50]. Secondly, we observed that both research teams used relatively low doses of vitamins compared to the regimens of other RCTs [48,50]. Therefore, these reports require further investigation, especially in light of the findings arising from some observational studies. They revealed that lower vitamin C and E concentrations corresponded with endometriosis-associated infertility, and the authors explained this association by comparing the consumption of these vitamins to the needs of antioxidant defense [51,52].

Other interesting observations regarding the role of antioxidant vitamins in endometriosis were presented in animal models; however, the authors analyzed their actions separately. Vitamin C, in addition to suppressing the growth of endometriosis lesions [53,54,55], has been mentioned as a vitamin intensifying the apoptosis [56] and degeneration of atretic follicles [53]. Similarly, the beneficial properties of vitamin E were shown in the study by Ni et al. using a mouse model. They indicated the capability of vitamin E to improve fertility directly via the restoration of antioxidant predominance disturbed by iron accumulation [57]. Taking into consideration these promising observations drawn from animal studies, attention should be paid to looking for novel mechanisms of antioxidant vitamins’ actions.

### 3.2. Micronutrients

#### 3.2.1. Zinc

Zinc is an antioxidant player exhibiting its function through the following twin-track action. Besides being a component of copper/zinc superoxide dismutase (Cu/Zn-SOD), an enzyme participating in free radical disposal, this micronutrient can directly display such an effect as a metal [58,59,60]. The observed tendency for reduced serum zinc concentrations, as well as a lower zinc intake among women suffering from endometriosis in comparison to healthy controls, allows us to conclude the protective effects of this micronutrient against endometriosis occurrence [49,61,62]. However, only the study led by Singh et al. suggested that zinc deficiency may be crucial in endometriosis-related infertility. The authors presented two observations: firstly, follicular fluid zinc concentrations were lower in patients with infertility and endometriosis compared to patients with tubal infertility, and secondly, follicular fluid zinc concentrations were positively correlated with the success of IVF procedures in patients with endometriosis [32]. These links can be explained by the complex effects of zinc in the maintenance of the normal functioning of oocytes. Thus, it was found that the deficiency of this micronutrient promotes a wide range of biological implications shared by ROS excess, including mitochondrial alterations or enhanced apoptosis in oocytes [63].

#### 3.2.2. Selenium

Selenium is an extremely essential micronutrient that participates in multiple processes that are crucial for homeostasis maintenance. The positive role of this trace element takes place through its involvement in the structure of several enzymes known as selenoproteins, including GPx, thioredoxin reductase (TrxR), and iodothyronine deiodinase (Dio) acting predominantly as antioxidant defenses [64,65]. In general, the link between selenium-containing enzymes and proper reproductive functions is well established, and selenium deficiency is known to be responsible for many causes of female fertility impairment [65]. Unfortunately, the role of this micronutrient in fertility problems in patients with endometriosis has been scantily discussed. Singh et al. observed that in infertile women with endometriosis, the concentrations of selenium and GPx measured in follicular fluid tended to be significantly lower in comparison to the concentrations in the group with tubal infertility. This association suggests that selenium deficiency may be one of the factors triggering the occurrence of infertility in patients with endometriosis. On the other hand, this study also did not find a link between selenium levels and pregnancy outcomes in women with endometriosis [32]. This observation stays slightly in contradiction with the data from the current literature, based on which it can be hypothesized that lower values of selenium affect folliculogenesis and oocyte development. In general, higher follicular fluid concentrations of selenium were related to better folliculogenesis and IVF outcomes [66,67]. Therefore, the detailed role of this micronutrient in endometriosis-associated infertility needs to be further investigated to prove the reasonableness of its supplementation and propose therapeutic doses.

### 3.3. Curcumin

Curcumin is a polyphenol contained in turmeric, and it is characterized by many pro-healthy properties of which its antioxidant potential deserves significant attention. The bioavailability of this compound is variable; however, in dietary supplements, these limitations could be eliminated to some extent [68,69]. Although studies regarding the influence of curcumin supplementation on infertility in human individuals with endometriosis are lacking, the increasing lines of evidence resulting from in vitro and animal studies suggest the high utility of curcumin in the management of endometriosis-related infertility. Most reports have focused on curcumin’s role in disease progression. Curcumin displayed the capability to reduce endometriosis lesions [70,71,72,73,74,75,76] as well as to mitigate adhesion formation [70,76]. In addition, the whole range of processes occurring at the cellular level was found to reflect the aforementioned action. After the application of curcumin, the reduced proliferation [73,77,78] and adhesion of ectopic endometrial cells [77], as well as enhanced apoptosis, were observed [75,78,79]. These biological changes were conditioned by alterations in the expressions of VEGF, matrix metalloproteinases (MMPs), and many other biomolecules that are mediators of inflammation, including TNF-α or pro-inflammatory interleukins (ILs). Since all of these biomolecules are involved in a complex cascade of oxidative status regulation [80,81,82], it can be indisputably assumed that curcumin affects endometriosis development by influencing this pathway [70,72,75,77,79,83,84,85,86].

Interestingly, two research teams observed that curcumin contributed to the enhancement of OS in endometriosis models; however, it was concluded that it was an initial effect that occurred as a result of OS defense [74,78]. These observations suggest the legitimacy of long-lasting curcumin supplementation. In line with the hypothesis regarding the antioxidant properties of curcumin are the findings by Swarnaker and colleagues. They found that the beneficial antioxidant effect of curcumin is based on its protective action against lipid and protein peroxidation [72]. Similarly, Ding et al. observed that curcumin supplementation alleviates pyroptosis, which is tantamount to efficient oxidant defense [76]. The only research that focused on the changes in the functioning of oocytes incubated in endometriosis-like conditions exposed to curcumin also noticed an association between such supplementation and OS-related improvement of oocyte functioning. It was proven that under high curcumin concentrations, the TNF-α values decreased, which contributed to improved folliculogenesis [87].

### 3.4. Melatonin

Melatonin, in addition to being a hormone regulating the circadian rhythm, due to its anti-inflammatory properties and participation in the elimination of oxidative imbalance, acts as a desirable supplement [88,89]. Unfortunately, most of the studies focused on the role of melatonin in endometriosis were conducted on animals or on in vitro models. Nevertheless, based on their results, some valuable conclusions can be formulated. As shown in Table 2, melatonin, regardless of its supplementation dose and administration route, has displayed a favorable effect on the attenuation of the endometriosis progression in animal models. Although the appropriate interpretation and transferring of the results from mice to humans are necessary, it can be speculated that even small doses of melatonin will cause the expected effects. Most researchers have observed a reduction in endometriotic lesions [33,90,91,92,93,94,95]. Although Mosher et al. suggested that the particular importance of melatonin in alleviating endometriosis seems to be due to the substance’s effect on estrogen-dependent alterations accompanying disease [96], according to many reports, melatonin is also able to affect oxidative status. So far, several studies have indicated melatonin as a substance directly influencing oxidative balance by lowering MDA levels, as well as improving CAT and SOD concentrations, which allows it to be perceived as an oxidative defense tool [92,93,95,97]. Noteworthy results in terms of the association between the antioxidant properties of melatonin and fertility were formulated by Lin et al. [33]. They revealed that melatonin reduced the whole range of OS-induced processes. First, in the cellular model, melatonin attenuated the expressions of such molecules as SA β-gal, GRP78, pIRE1, CHOP, p16, p21, and p-H2AX, and it was able to stimulate apoptosis, enhance the ATP levels, alter MMP production, and increase the expressions of SOD-1 and inducible nitric oxide synthase (iNOS), which resulted in the lowering of granulosa cell senescence. Secondly, they confirmed these changes using a mouse model and linked them with better reproductive outcomes in supplemented animals [33]. The antioxidative potential of melatonin was also revealed by Paul et al.’s research team, which, in two studies, found that this substance strongly affected MMP secretion and alleviated the oxidation of lipids and proteins in mice with induced endometriosis [98,99].

### 3.5. N-Acetylocysteine (NAC)

N-acetylcysteine (NAC) is an agent exhibiting antioxidant properties through the maintenance of glutathione action. It has been applied in many areas of health from the treatment of all oxidative imbalance-related conditions to the management of various psychiatric disturbances [100,101]. NAC was also found to successfully support female fertility; however, these reports concerned patients with various causes of infertility [23].

So far, three prospective studies investigating the influence of NAC on endometriosis development and fertility outcomes, including only the patients suffering from endometriosis, have been conducted. Anastasi et al. led a study that included 120 patients with endometriosis who were supplemented with 1.8 g of NAC administrated in three divided doses, three consecutive days a week, for three weeks. Such supplementation resulted in pregnancy in 86.5% of patients who previously wanted to conceive. Thus, the results are highly encouraging, and their only limitation is the fact that it is unknown what percentage of women were diagnosed with infertility [102]. Another study led by Porpora et al. did not find a statistically significant difference in the pregnancy rate between a group of 47 patients supplemented with NAC at the same dosage schedule as in the aforementioned study and a group of 45 non-supplemented patients from the control group. Nevertheless, the authors concluded that the influence of NAC on fertility outcomes was beneficial, considering other effects of its intake and the lack of fertility impairment [103]. These two studies also consistently observed that NAC had a positive impact on the inhibition of endometriosis development. The authors noticed that supplementation with NAC resulted in a lower size of the endometrial cyst [102,103], a reduced volume [103], and a lower number of lesions [103]. Slightly contrasting observations were revealed in the randomized controlled trial by Asgari et al. They found that NAC did not minimize the ovarian endometrioma recurrence risk. Nevertheless, these inconsistencies can be explained by the fact that the group supplemented with NAC, similarly to the control group, was also treated with oral contraceptives and that the endometrial lesions were previously radically removed. Such a combination of therapies together with the excision of endometriosis tissue may incompletely reveal NAC properties [104]. 

The beneficial effects of NAC supplementation have also been demonstrated in animal models. Supplementation with NAC in animals in which endometriosis was induced contributed to lower COX-2, MMP-9, and TNF-α expression, which are all molecules participating in OS enhancement [105,106].

### 3.6. Coenzyme Q10 (CoQ10)

Coenzyme Q10 (CoQ10), also known as ubiquinone, is a molecule constituting an important part of cell and mitochondrial membranes, which renders it responsible for the proper course of the intracellular respiratory process. In addition, CoQ10 is present in free form in the cytoplasm, where it acts as a protective factor against the oxidation of lipids or proteins [107,108]. So far, only two studies have evaluated the role of CoQ10 in endometriosis [109,110].

In rats with experimentally induced endometriosis, supplementation with CoQ10 resulted in lower lesion volumes, decreased the histopathological score, decreased adhesion formation, decreased the activities of VEGF and MMP-9, and enhanced apoptosis. All of these changes indicate the inhibition of endometriosis progression [109]. Another study found that CoQ10 seems to have a beneficial effect on the conditions of animal oocytes incubated in the follicular fluid obtained from women with endometriosis. Such therapy resulted in the reduced risk of aberrations within oocytes’ spindles and, consequently, in the improvement of oocytes’ functioning [110].

According to the study by Govatati et al., endometriosis has been linked to genetic alterations within genes encoding mitochondrial membrane complex-1 (MMC-1) built with NADH and ubiquinone. Nevertheless, these results indirectly indicate the importance of CoQ10 in endometriosis, and the potential effects of its supplementation on the course of the disease require further investigation [111].

### 3.7. Resveratrol

Resveratrol belongs to the family of polyphenols and is a naturally occurring compound in many plant-based food products. It can also be synthetically produced using new processing methods. The simplicity of obtaining resveratrol together with its many health-enhancing properties render it a valuable supplement revealing strong antioxidant properties [112,113]. However, considering the use of resveratrol as a supplement in patients with endometriosis, the slightly different mechanisms of action displayed by the different forms of this substance on endometriosis cells should be taken into account [114]. 

Three studies assessing the impact of resveratrol supplementation on eutopic endometrium in patients suffering from endometriosis seem to be the most crucial in evaluating the influence of treatment with resveratrol on female fertility [115,116,117].

Two randomized controlled trials conducted by the same research team were conducted on the same patient groups involving 34 women with endometriosis-associated infertility. Among them, 17 were treated with 800 mg of resveratrol divided into two equal doses that were administrated for 12 to 14 weeks together with oral contraceptive pills added for the last three weeks of this experimental therapy, and 17 were non-supplemented individuals. Both before and after the treatment, the eutopic endometrial tissue fragments were collected and taken under investigation [115,116]. The first study revealed that resveratrol supplementation contributed to lower MMP-9 expression [115]. In turn, another study noticed that women who had taken resveratrol exhibited lower endometrial TNF-α2 and VEGF concentrations [116]. Importantly, since all of these above-mentioned molecules were described as factors deteriorating reproductive outcomes [118,119,120], resveratrol seems to play an important role in the regulation of eutopic endometrium functioning and help to improve its quality. Another study led by Maia et al. found that resveratrol at a dose of 30 mg combined with oral contraceptive pills resulted in decreased COX-2 and aromatase endometrial expressions in comparison to an oral contraceptive pill treatment alone [117]. Such supplementation may lead to the inhibition of disease progression through there being a lower capacity of endometrial cells to form new ectopic lesions [121,122]. Although the results of these studies indicate that resveratrol has positive effects, it is worth noting that resveratrol was not supplemented alone, but always in combination with oral contraceptive pills. Hence, there is a risk that the oral contraceptive pills strengthened the effects of resveratrol, and future studies should evaluate the effects of resveratrol without additional substances.

In addition, numerous studies conducted on the animal and in vitro models have shown that resveratrol suppresses endometriosis development. Researchers consistently found that resveratrol supplementation in rodents with experimentally induced endometriosis hindered the endometriosis lesions’ progression, enhanced apoptosis, as well as redirected the immune response toward anti-inflammatory and antioxidative actions [123,124,125,126,127,128,129]. Similarly, in endometriosis cell-based models, resveratrol exerted analogous effects [125,130,131,132,133]. Only the recently published study by Zou et al. raised doubts on the antioxidative effects of resveratrol in endometriosis. The authors found that a lower proliferation and cell migration were related to enhanced ferroptosis associated with an exacerbated occurrence of the OS state [134].

## 4. Conclusions

Understanding the substantial role of oxidative imbalance in the pathogenesis of endometriosis enabled the exploration of antioxidant usage in supportive endometriosis treatment. Antioxidants show promise as agents for improving fertility in patients with endometriosis. Numerous animal and in vitro model studies revealed the ability of antioxidants to inhibit ectopic lesion progression and demonstrated changes in the whole range of substances, which reflect the oxidative state of the organism. The results from several clinical trials involving women with endometriosis also present reductions in different markers of oxidative stress; however, data regarding the direct influence on pregnancy are still limited. As most studies have been conducted on animal and in vitro models, there is a great need for the design of RCTs involving human individuals with endometriosis.

## Figures and Tables

**Table 1:** A summary of the results of randomized controlled trials evaluating the effects of supplementation with vitamins displaying antioxidant potential in women with endometriosis.

Authors	Year of Study	Type of Study	Study Groups	Supplemented Vitamins	Dose	Effects of Supplementation
Amini et al. [46]	2021	randomized controlled trial	60 patients with endometriosis divided into 2 groups: group 1—30 patients supplemented with vitamins C and E; group 2—30 patients without supplementation	combination of vitamin C and vitamin E	vitamin C at a dose of 1000 mg/day in two equal doses and vitamin E at a dose of 800 IU/day in two equal doses for 8 weeks	-lower serum malondialdehyde (MDA) and reactive oxygen species (ROS) levels
Santanam et al. [47]	2013	randomized placebo-controlled trial	59 patients with endometriosis divided into 2 groups: group 1—46 patients supplemented with vitamins C and E; group 2—13 patients without supplementation	combination of vitamin C and vitamin E	vitamin C at a dose of 1000 mg/day in two equal doses and vitamin E at a dose of 1200 IU/day in three equal doses for 8 weeks	-lower levels of interleukin 6 (IL-6), regulated on activation, normal T-cell expressed and secreted (RANTES), and monocyte chemoattractant protein-1 (MCP-1) in peritoneal fluid
Mier-Cabrera et al. [48]	2008	randomized controlled trial	34 patients with infertility and endometriosis divided into 2 groups: group 1—16 patients supplemented with vitamins C and E; group 2—18 patients without supplementation	combination of vitamin C and vitamin E	vitamin C at a dose of 343 mg/day and vitamin E at a dose of 84 mg/day for 6 months	-lower serum MDA and lipid hydroperoxide (LOOH) levels
Mier-Cabrera et al. [49]	2009	randomized controlled trial	72 patients with endometriosis divided into 2 groups: group 1—37 patients supplemented with vitamins C, E, and A; group 2—35 patients without supplementation	vitamins C, E, and A	vitamin C at a dose of 500 mg/day, vitamin E at a dose of 20 mg/day, and vitamin A at a dose of 1050 μg/day for 4 months	-higher serum levels of vitamin E, vitamin C, and retinol -increased serum superoxide dismutase (SOD) and glutathione peroxidase (GPx) activity -decreased serum MDA and lipid hydroperoxide (LPH) levels
Lu et al. [50]	2018	randomized controlled trial	280 infertile patients with endometriosis divided into 2 groups: group 1—160 patients supplemented with vitamin C; group 2—120 patients without supplementation and 150 controls without endometriosis	vitamin C	vitamin C at a dose of 1000 mg/day for 8 weeks	-higher follicular fluid and serum vitamin C levels

**Table 2:** Tabular summary of melatonin supplementation in animal models.

Authors	Year of Study	Study Groups	Supplementation Dose, Period of Supplementation, and Methods of Melatonin Administration	Effects of Melatonin Supplementation
Lin et al. [33]	2020	84 female mice	1.5 mg per mouse per day for 2 weeks; intraperitoneal administration	-smaller size of endometriotic lesions -increased number of pups -lowered expressions of GRP78, p-IRE1, CHOP, p16, p21, and pH2AX and enhanced expressions of superoxide dismutase 1 (SOD1) and inducible nitric oxide synthase (iNOS) in ovaries
Park et al. [90]	2023	20 female mice	50 mg/kg/day for 2 weeks; oral administration	-lower volume, weight, and growth of endometriotic lesions -lower expressions of Ccnd1, Ccne1, and Pcna mRNAs in endometriotic lesions
Kocadal et al. [91]	2013	22 female rats	20 mg/kg/day for 2 weeks; intramuscular or intraperitoneal administration	-lower volume of endometriotic lesions
Yildirim et al. [92]	2010	23 female rats	10 mg/kg/day for 2 weeks; intraperitoneal or subcutaneous administration	-lower volume of endometriotic lesions -lower histopathological score -higher SOD and catalase (CAT) levels and lower malondialdehyde (MDA) levels
Güney et al. [93]	2008	25 female rats	10 mg/kg/day for 4 weeks; intraperitoneal administration	-lower volume and weight of endometriotic lesions -lower histopathological score -lower cyclooxygenase 2 (COX-2) expression and MDA levels -higher SOD and CAT levels
Cetinkaya et al. [94]	2015	32 female rats	10 mg/kg/day or 20 mg/kg/day for 2 weeks; intramuscular or intraperitoneal administration	-lower volume of endometriotic lesions
Yilmaz et al. [95]	2015	20 female rats	10 mg/kg/day for 4 weeks; intraperitoneal administration	-lower volume and weight of endometriotic lesions -lower histopathological score -higher SOD levels -lower MDA levels -lower vascular endothelial growth factor (VEGF) score -higher tissue inhibitor of metalloproteinase 2 (TIMP-2) score and lower matrix metalloproteinase 9 (MMP-9) score
Yesildaglar et al. [97]	2016	30 severe combined immunodeficient (SCID) female mice	20 mg/kg/day for 4 weeks; subcutaneous administration	-lower MDA levels -lower histopathological score
Paul et al. [98]	2008	79 female mice	48 mg/kg/day for 20 days; intraperitoneal administration	-lower proMMP-9 activity and enhanced expression of TIMP-1 -lower protein oxidation and lipid peroxidation
Paul et al. [99]	2010	24 female mice	16, 32, or 48 mg/kg administered intraperitoneally (i.p) twice daily for 3 days before endometriosis induction and further intraperitoneal administration at 48 mg/kg per day for 10 or 20 days	-lower tumor necrosis factor α (TNF-α) expression -lower expression of MMP-3 mRNA and higher expression of TIMP-3 -enhanced apoptosis mediated through weakened Bcl2 expression and induced Bax expression

## Data Availability

Not applicable.
